# Healthcare Professionals’ Knowledge, Attitudes, and Practices in Providing Care to Southeast Asian Immigrants with Cardiometabolic Syndrome: A Scoping Review

**DOI:** 10.1007/s40615-024-02129-3

**Published:** 2024-08-20

**Authors:** Diane Gargya, Kathy Nguyen, Ieva Stupans, Thilini Thrimawithana, Vincent Chan, Karen Livesay, Barbora de Courten, Chiao Xin Lim

**Affiliations:** 1 Pharmacy, School of Health and Biomedical Sciences, RMIT University, Bundoora, VIC 3083 Australia; 2 Medicine Department, School of Clinical Sciences, Monash University, Clayton, VIC 3168 Australia; 3 Nursing, School of Health and Biomedical Sciences, RMIT University, Bundoora, VIC 3083 Australia; 4 School of Health and Biomedical Sciences, RMIT University, Bundoora, VIC 3083 Australia

**Keywords:** Reduced inequalities, Knowledge, attitudes, and practices (KAP), Healthcare professionals, Southeast Asian immigrant, Cardiometabolic healthcare delivery, Co-production

## Abstract

**Objective:**

There is a growing emphasis on healthcare professionals’ (HCPs) role in managing cardiometabolic risk factors to reduce health disparity for immigrants in developed countries. This scoping review aimed to analyse evidence about HCPs’ knowledge, attitudes, and practices (KAP) of managing cardiometabolic risk factors among Southeast Asian (SEA) immigrants in developed countries.

**Design:**

Primary studies from inception to July 17, 2023, from four databases: PubMed/Medline, Embase, PsycINFO, and CINAHL were included. This review followed the Joanna Briggs Institute (JBI) scoping review methodology and reported in line with PRISMA-ScR.

**Results:**

Of 619 identified studies, seven met the inclusion criteria. All studies discussed HCPs’ knowledge, six explored attitudes, and three described practices specific to SEA immigrants. The extracted data were analysed using descriptive qualitative content analysis and classified into barriers and facilitators. Barriers included cultural discordance and acculturation challenges (patient level); gaps in cultural understanding, communication and clinical skills (healthcare team level); limited immigrant-specific resources (organisation level); and funding constraints (environment level). Facilitators included community and provider support (patient level), awareness and desires to provide immigrant-specific care (healthcare team level), availability of culturally appropriate services (organisation level), and multicultural agendas and policies (environment level).

**Conclusion:**

The barriers and facilitators faced by HCPs caring for SEA immigrants with cardiometabolic syndromes share similarities with other immigrant groups. Future research focused on co-production involving immigrant patients, their communities, and HCPs in healthcare service design is required to support HCPs in providing culturally appropriate care and promoting health equity regardless of ethnic, cultural, or linguistic backgrounds.

**Supplementary Information:**

The online version contains supplementary material available at 10.1007/s40615-024-02129-3.

## Introduction

The World Health Organisation (WHO) World Report on the Health of Refugees and Migrants, published in 2022, identified that immigrant populations have an increasing health burden of non-communicable diseases, including type 2 diabetes, hypertension, and cardiovascular diseases [1]. The development of these conditions is progressive. Following the International Diabetes Federation’s definition, cardiometabolic syndrome (or metabolic syndrome) is characterised by a cluster of metabolic abnormalities such as central obesity (measured by waist circumference), impaired glucose metabolism (impaired fasting glucose or type 2 diabetes), dyslipidaemia (high serum triglyceride or low serum high-density lipoprotein cholesterol concentrations), and increased blood pressure [2]. Immigrants in this review follow the International Organisation for Migration definition as ‘a person who moves into a country other than that of his or her nationality or usual residence so that the country of destination effectively becomes his or her new country of usual residence’ [3].

The large-scale population movements in the region of Southeast Asia (SEA) — Brunei Darussalam, Cambodia, Indonesia, the Lao People’s Democratic Republic (PDR), Malaysia, Myanmar, the Philippines, Singapore, Thailand, Timor-Leste, and Vietnam — have been happening since European colonisation to post-Second World War [4]. It is estimated that 23.6 million SEA people live outside their country of birth, for example, in Australia, Canada, New Zealand, and the USA [4]. The country of destination is considerably influenced by existing social networks, opportunities, and freedom/safety [5, 6]. A 2015 report shows that SEA immigrants in Australia were 10–20% more likely to report being treated for hypertension and/or dyslipidaemia compared to Australian-born participants [7], while a 2014 study reported that in the USA, the risk of dyslipidaemia was higher among SEA immigrants, including Filipino and Vietnamese women compared with non-Hispanic whites [8]. Early research on gene-diet interaction has shown that specific dietary practices among SEA populations can exacerbate or mitigate genetic predispositions to diabetes and cardiovascular conditions [9]. As the diaspora of SEA people continues to increase, it is projected to contribute to population growth in high-income countries [10]. Examining SEA immigrant populations’ access and utilisation of healthcare services in their host countries and their health disparities is crucial. It will enable health organisations in host countries to address SEA communities’ unique challenges to provide equitable care and improve cardiometabolic health outcomes.

Ethnic health burdens and disparities are complex and involve multiple factors, with social determinants of health playing a crucial role [1]. These determinants, which exist outside of the healthcare delivery system, can significantly impact the treatment and management of cardiometabolic syndrome [1, 11–13]. Immigrant patients often face challenges in effective communication, including language proficiency and adapting to the host country’s cultural norms [12, 13]. Additionally, various cultural factors, including health beliefs, behaviours, religions, and family or community dynamics, can affect engagement with healthcare services and health management [14–16]. These factors underscore the need for structured therapeutic lifestyle changes and engagement in patient self-care, including a healthy diet and regular physical activities that are adapted to the patient’s cultural needs [2]. Furthermore, the patient’s engagement in their health management is impacted by how therapeutic and lifestyle information is communicated and the nature of the patient-provider interactions [14].

Previous studies on HCPs’ providing care to immigrant patients evaluated their knowledge and competencies to work with immigrant patients as moderate (64.2%) [17], while in another study, approximately 80% were satisfied with their level of cultural knowledge, even though many had not received formal training [18]. More than half (61%) of health workers have a positive attitude towards immigrant patients’ interaction with health services [17], although HCPs’ attitudes towards immigrant patients were scarcely acknowledged in practice [18]. Studies show that while there are improvements in knowledge and attitudes post-training, these do not always lead to significant changes in the HCPs’ practice and organisation [18–20]. Knowledge of social determinants, legal and policy contexts, and specific health differences among migrant populations allows HCPs to better understand and address these patients’ unique challenges [12, 21, 22]. Positive attitudes, such as respect, empathy, and cultural sensitivity, foster trust and improve patient-provider relationships [21–23]. Pragmatic skills or practices, including effective communication and the ability to work with interpreters, ensure that HCPs can meet the individual needs of migrant patients [12, 21, 22]. HCPs need to act as advisors and collaborators, effectively communicate, and develop individualised self-management plans with the patient [14]. Thus, the knowledge, attitude, and practice (KAP) of HCPs are crucial for providing effective healthcare to migrant and minority patients and delivering culturally sensitive care [21]. Still, a gap exists in the real-life implementation of needed lifestyle changes, particularly in cardiometabolic syndrome management, including diabetes [14], particularly among immigrant patients from SEA.

Although cardiometabolic risk factor modification mainly occurs during interactions between patients and HCPs, the effectiveness of any healthcare quality improvement initiative, prevention, or disease management approach is thought to hinge on the interdependent relationship of different levels of stakeholders — (1) the patient; (2) the healthcare team (e.g. HCPs, family members and others); (3) the organisation or infrastructure/resources; and (4) the environment or regulatory, market, and policy framework to maximise the probability of improving the quality of healthcare [24, 25]. The goal at the environmental and organisation-wide level is to provide an optimal setting that includes policies and financial support for providing healthcare for cardiometabolic syndromes. The healthcare disparities of the immigrant populations are compounded by decreased healthcare accessibility and the inability to identify or adequately address populations at risk [12, 13]. Therefore, a holistic approach to cardiometabolic care provision of HCPs using the ‘Four-Level Model of Healthcare System’ framework to present the findings [24] can provide a system-wide view of the HCPs’ KAPs in providing care to SEA immigrant patients in developed countries’ healthcare system [12].

Previous reviews on immigrant care have primarily focused on patient perspectives and engagement [19, 22, 26–30], while those examining healthcare professionals’ experiences [12, 31–34] have not explicitly addressed cardiometabolic care provision to SEA immigrants [12, 13]. Considering the growing emphasis on the role of HCPs in the management of cardiometabolic syndromes and the increasing health disparity among SEA immigrants, this scoping review may be the first attempt to map the research done in this area, as well as to synthesise the available evidence. This scoping review was guided by the research question, ‘What barriers and facilitators exist to HCPs’ knowledge, attitudes, and practices regarding the provision of cardiometabolic syndrome healthcare to SEA immigrants in developed countries?’.

## Methods

### Study Design

A scoping review using the JBI methodology [35] was selected to explore available academic literature and map the evidence on HCPs’ knowledge, attitudes, and practices in providing cardiometabolic syndrome care to SEA immigrants. This review is reported using the PRISMA-ScR checklist [36]. The scoping review protocol was registered with the Open Science Framework Registries (https://osf.io/j9wpf).

### Inclusion Criteria

The search strategy (Table 1) was guided by the Population, Concept, and Context model [37]. Relevant studies were searched using the following databases: PubMed/Medline, Embase, PsycINFO, and CINAHL from inception to July 17, 2023. The search strategy was reviewed and modified by DG, CL, VC, IS, and TT in an iterative process to ensure an optimal extraction of relevant articles. The search was expanded to include article titles, abstracts, keywords, and full text without limiters as applicable to each database (Appendix 2). The main search was supplemented with hand searching of included articles’ references list. Hand searches were also conducted using Google Scholar’s ‘cited by’ and ‘related articles’ functions [38], and ResearchRabbit’s visualisation map of ‘similar work’ and ‘all citations’ [39] of each included article.

**Table 1:** Search strategy using the population, concept, and context model

Population	‘health* personnel’ OR ‘health personnel’ OR ‘primary healthcare’ OR ‘health professional’ OR ‘healthcare provider’ OR ‘health practitioner’ OR diet* OR nutritionist OR pharmac* OR dent* OR physician* OR clinician OR ‘general practitioner’ OR doctor OR nurse OR optometr* OR podiatrist OR therapist OR ‘diabetes educator’ OR psycho* OR ‘family practice’ OR ‘multidisciplinary care team’ OR ‘health care’ OR ‘primary care’ OR ‘shared services’ OR ‘medical interpreter’ OR patient* OR consumer*
Concept	(knowledge OR attitude OR practice OR ‘health service’ OR ‘health care delivery’ OR ‘delivery of health care’ OR ‘quality care’ OR ‘patient-centred care’ OR ‘patient-centered care’ OR ‘shared decision-making’ OR ‘dietetic care’ OR communication OR experience OR view* OR opinion OR perception OR belief OR perspective OR strateg* OR approach) AND (hypertension OR ‘high blood pressure’ OR ‘elevated blood pressure’ OR hypertensive OR ‘high cholesterol’ OR ‘high sugar’ OR ‘cardiometabolic risk factors’ OR ‘metabolic syndrome’ OR diabetes OR prediabet* OR ‘impaired glucose tolerance’ OR ‘impaired fasting glucose’ OR ‘insulin resistance’ OR ‘abdominal obesity’ OR ‘cardiovascular disease’ OR ‘cardiac disease’ OR ‘cardiovascular health’ OR ‘heart disease’ OR ‘heart condition’ OR ‘heart disease risk factors’ OR hyperlipidemia OR hyperlipidaemia OR dyslipidemia OR dyslipidaemia OR hypercholesterol*emia OR ‘lipid disorder’)
Context	(‘southeast asia*’ OR ‘south east asia*’ OR cambodia* OR indonesia* OR laos OR laotian OR malaysia* OR singapore* OR filipino OR Philippines OR thai* OR vietnam* OR burm* OR myanma* OR brunei*) AND (immigrant OR migrant OR emigrant OR refugee OR ‘ethnic minorit*’ OR ‘vulnerable population’ OR ‘minority group’ OR ‘health minorit*’ OR ‘culturally and linguistically diverse’ OR CALD)

Studies were eligible for inclusion if they were primary studies reporting the care of adult SEA immigrants living in developed host countries based on the United Nations classification including Australia, Canada, UK, and United States of America [40], without any restriction of the healthcare setting, and published in the English language. All study designs were included.

For this review, SEA people were those who were born from any of the member countries of the Association of Southeast Asian Nations (ASEAN) — Brunei Darussalam, Burma, Cambodia, Indonesia, Lao PDR, Malaysia, Philippines, Singapore, Thailand, and Vietnam [41]. By these definitions, SEA immigrants were individuals who originally came from any of the Southeast Asian countries to live permanently in a host country [3].

HCPs included in the study were those with an academic education, e.g. medical doctors, nurses, and pharmacists, practising in developed countries and involved in caring for adult (18 years of age or older) SEA immigrants with cardiometabolic syndrome.

### Exclusion Criteria

Studies on migrant/foreign workers and undocumented migrants were excluded [3]. The review also excluded non-health professional carers, e.g. family members and case workers, for whom health management was beyond the scope of formal health management services.

### Study Selection Process

EndNote (Clarivate, USA) software was used in the screening process [42]. Two authors (DG and KN) independently performed a two-stage selection process. The initial stage involved screening the title and abstract of the extracted articles, and in the second stage, full-text screening was undertaken. A third author (CL) resolved any disagreement during the screening process.

### Data Extraction

A data extraction form was created using MS Excel (Microsoft Corporation, USA) spreadsheet to collect author names, article titles, year of publication, participant characteristics (profession, gender, age, etc.), study aims, study design, location, sample size, phenomenon of interest, data collection, and findings. The data extraction tool was pilot-tested on two articles included by one author (DG) and reviewed by research team members (VC, IS, TT, CL). Information from the remaining studies was extracted by the same author and verified by another author (KN). Only data pertaining to HCPs were collected for analysis.

### Synthesis

Results were summarised using descriptive qualitative content analysis [43] guided by the ‘Four-level Model of Healthcare System’ [24], highlighting the importance of recognising the interdependence of the different levels. Themes from the extracted HCP data were coded through open coding and classified into the different levels of the healthcare system based on the involvement of the patient, healthcare team, organisation, or the environment. Themes were grouped into a broader order of main categories, generic categories, and sub-categories. The themes were also classified into HCP knowledge, attitudes, or practices and further categorised into barriers and facilitators. Data was analysed and synthesised by one author (DG) and verified by another author (KN), with ongoing discussions among the research team.

## Results

### Selection of Sources of Evidence/Search Results

**Fig. 1:**
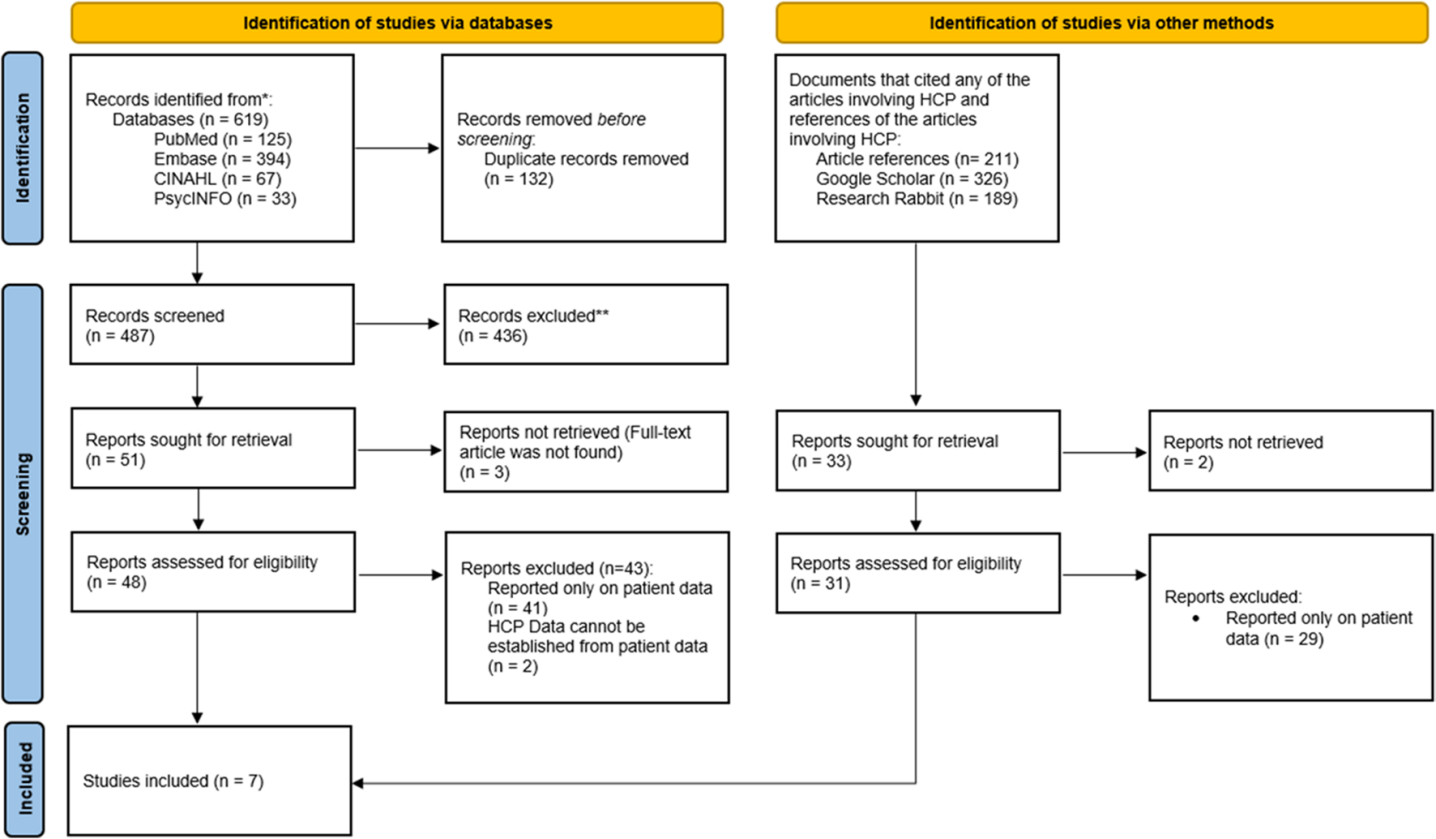
PRISMA flow diagram of articles screened for scoping review

The database search and screening initially identified 619 articles (Fig. 1). After 132 duplicate articles were removed, 487 articles were screened through title and abstract screening. Forty-eight articles were eligible for full-text review. The majority of the articles (*n* = 43) were excluded as the knowledge, attitudes, and practices of HCPs were not included in the study design. Two additional papers were identified by hand-searching references and study citations. A total of seven articles were included in this review.

### Description of Included Studies

All studies were of qualitative design and involved both patients and HCPs [44–50]. Most studies employed interviews for data collection [40–49] and thematic analysis for data interpretation [44–47]. Additional analyses included content [48] and subjective/interpretative approaches [49]. Three studies were guided by ethnographic methodology [44, 45, 47], and one study utilised focus groups with grounded theory for data analysis [50]. Five studies were undertaken in the USA [45, 46, 48–50], and two were conducted in Australia [44, 47]. None of the studies formally assessed HCPs’ KAPs; instead, they used guided interview questions regarding their experiences and views of providing care to patients with immigrant backgrounds.

The immigrant patients in the studies were of the following backgrounds: Cambodian (*n* = 1) [50], Vietnamese (*n* = 4) [45, 47–49], Filipino (*n* = 1) [46], and people of Chinese heritage from Cambodia, Malaysia, and Singapore (*n* = 1) [44]. Six of the included studies described diabetes management [44–48, 50] and three studies described the management of hypertension [45, 47, 49]. Doctors [44–50] and nurses [44, 46–50] were the primary HCPs interviewed within the community [44–50] and hospital settings [47]. Two studies recruited HCPs in their capacity as members of the Vietnamese [45] and Filipino communities [46]. The other three studies described interviews with HCPs of non-SEA ethnic backgrounds [47] and of Vietnamese ethnicity [39, 48]. Two studies did not identify the ethnic background of the HCPs [44, 50].

### Review Findings

**Fig. 2:**
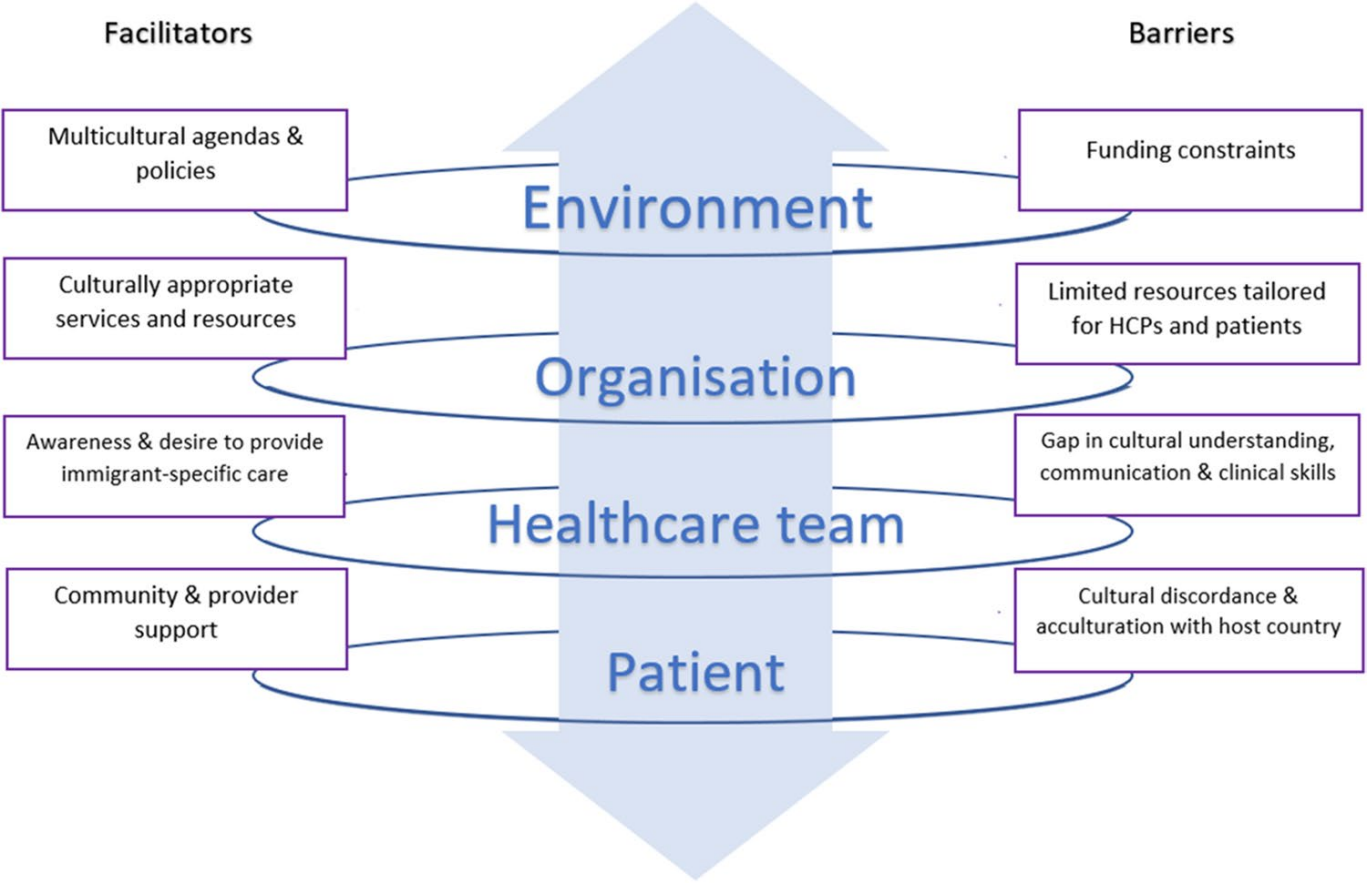
Barriers and facilitators in the HCPs’ practice in providing healthcare to SEA immigrants against the 'Four-Level Model of Healthcare System'. (HCP, healthcare professional; SEA, Southeast Asian)

The HCP responses in the included studies were classified into categories reflecting knowledge, attitudes, or practices in providing cardiometabolic care to patients of SEA backgrounds. Due to the qualitative nature of the included studies, quantification of HCPs’ KAP was not possible. The studies that demonstrated KAPs of HCPs are instead summarised in Table 2. Themes from the included articles were presented using the modified ‘Four-Level Model of Healthcare System’ (Fig. 2) and further classified as barriers or facilitators of practice [12, 24, 25] (Tables 3 and 4).

**Table 2:** Summary of included studies

First author/year; location	HCP participants and practice setting	Service users and health condition(s) described*	Aim/objective	Data collection method with HCP; analysis and methodology	KAP domain
Choi, 2018 [44]; Australia (Sydney, New South Wales and Melbourne, Victoria)	*n* = 8 (dieticians, diabetes nurse educators, endocrinologists, bilingual health educators); Community diabetes health services	Chinese from Malaysia, Singapore, Cambodia, China, and Hong Kong; type 2 diabetes	To understand the experience of Chinese migrants living with type 2 diabetes in Australia and explore their culturally specific diabetes management needs, habits, and expectations in the Australian context to help shape an Australian Chinese diabetes service	In-person in-depth interviews; thematic analysis with pattern matching; ethnographic data collection	Knowledge and attitudes
Chu, 2011 [45]; US (Oklahoma)	*n* = 6 (medical doctor, dentist, pharmacist, and nutritionist); community leaders and healthcare providers of a Vietnamese community	Vietnamese; hypertension, diabetes, cancer, dementia, depression	To describe the barriers and facilitators to healthcare access and utilisation for Vietnamese-American elders in a medium-sized urban area in the Midwest	In-person interview, field notes and researcher observation; ethnographic analysis	Knowledge and attitudes
Finucane, 2008 [46]; US (Hawaii)	*n* = 7 (medical doctor, registered nurse, public health academic, anthropology academic, cultural leader); community health and cultural experts	Filipino; type 2 diabetes	To identify the cultural values, traditions, and perceptions of diabetes risk and self-care among Filipino Americans in Hawaii with type 2 diabetes that facilitate or impede engagement in diabetes self-management behaviours and education classes	In-person, in-depth interviews; thematic analysis	Knowledge
Komaric, 2012 [47]; Australia (Queensland)	*n* = 14 (nutritionist, preadmission anaesthetist, medical doctor, nurse, audiologist, radiographer, occupational therapist, cardiac scientist, lifestyle program coordinator); Public and private hospital, community, and primary health services	Arabic-speaking, Chinese, Sudanese, Tongan and Vietnamese groups; hypertension, hypercholesterolaemia, type 2 diabetes	To describe the challenges people from CALD communities face regarding treating and preventing chronic disease and what barriers they experience and perceive regarding access to health services	Structured telephone interviews; thematic analysis	Knowledge, attitudes and practices
Mull, 2001 [48]; US (Orange County, California)	*n* = 8 (medical doctors, nurses, herbalists); community clinic serving the Vietnamese community	Vietnamese; type 2 diabetes	To describe the cultural context of type 2 diabetes mellitus among Vietnamese immigrants in the United States, including people’s ideas about cause and proper treatment, and to suggest ways in which better control of the disease can be achieved in this population	In-person semi-structured interviews, including opinions of patients’ interview responses; content analysis	Knowledge, attitudes and practices
Pham, 1999 [49]; US (Philadelphia, Pennsylvania)	*n* = 5 (medical doctors, registered nurse); Healthcare providers serving a substantial number of Vietnamese patients in the community	Vietnamese; hypertension, cardiovascular disease	To (1) assess the awareness and understanding of hypertension and cardiovascular disease and their perceived prevalence in the Philadelphia Vietnamese community, (2) define the healthcare problems, barriers, and needs of the community, and (3) characterise the community’s beliefs regarding Western medicine and folk medicine	In-person semi-structured interview; subjective/interpretative analysis	Knowledge and attitudes
Renfrew, 2013 [50]; US (Revere, Massachusetts)	*n* = 25 (medical doctors, nurses, dietician); multi-speciality community-based health centre	Cambodian; diabetes	To explore the potential barriers to care for Cambodian patients with diabetes who receive care at a low-income, predominantly immigrant, urban community health centre near Boston	In-person focus-group interview with discussion guide; Strauss and Corbin’s three-step approach to coding and analysis	Knowledge, attitudes and practices

^*^Health condition(s) described — patients’ health condition(s) as described in the primary study (*KAP*, knowledge, attitudes and practices; *CALD*, culturally and linguistically diverse)

**Table 3:** Identified barriers

Four-Level Model of Healthcare System	Theme (main category)	Sub-theme (generic category)	Code (sub-category)	References
Patient	Cultural discordance and acculturation with the host country	Health beliefs	• Discordance between Western medicine and patient’s health beliefs	[45, 47, 48, 50]
			• Dietary health beliefs and practices	[44, 46, 50]
		Cross-cultural communication barrier and limited literacy	• Limited literacy	[46–48, 50]
			• Communication challenges due to language barriers and cultural behaviours	[44, 47, 49, 50]
		Sociocultural and socioeconomic context	• Social and personal stigma of health conditions	[44, 46, 50]
			• Lack of patient self-sufficiency due to socioeconomic factors	[44, 47, 49]
			• Changes in family dynamics and responsibilities from immigration	[46, 50]
			• Negative impact of immigration from acculturation, loss of self-identity and lack of patient history	[45, 50]
			• Religious beliefs’ influence on health management	[46]
Healthcare team	Gap in cultural understanding, communication and clinical skills	Health beliefs	• Discordance between providers’ and patients’ health beliefs	[45, 47, 48, 50]
		Cross-cultural communication barrier	• Communication challenges due to language discordance and the additional resources and time to accommodate interpretation	[44, 47, 49, 50]
			• Immigrant HCP’s cultural behaviours influencing practice	[48]
		Clinical skills and cultural knowledge of the patient’s context	• Lack of appropriate training in the community’s culture and health care needs	[47, 48, 50]
Organisation	Limited resources tailored for HCPs and patients	Tailored resources to the needs of HCPs and patients	• Limited resources, including time and interpreting services	[47, 50]
			• Discordance between patient and service provider expectations	[44]
			• Accessibility and availability of services	[47]
Environment	Funding constraints	Funding	• Limited or a lack of financial support	[47–49]

**Table 4:** Identified facilitators

Four-Level Model of Mealthcare System	Theme (main category)	Sub-theme (generic category)	Code (sub-category)	References
Patient	Community and provider support	Family and community support	• Community’s confidence in Western medicine	[49]
		Encounters	• Family and community support with health services interactions	[46, 50]
			• Encounters with heath providers support patients’ health knowledge	[47]
Healthcare team	Awareness and desire to provide immigrant-specific care	Encounters	• Interactions between provider and patient support learning together	[47]
		Tailored resources and support	• Use of interpreter services and family member involvement	[46, 50]
		Sociocultural knowledge and awareness	• Knowledge of patients’ acculturation context and awareness of cultural incompatibility between health providers and patients	[47]
Organisation	Culturally appropriate services and resources	Tailored resources and support	• Availability of interpreter services and bilingual staff	[47, 49, 50]
		Community Partnership	• Community involvement and partnership in healthcare activities	[47]
		Cultural competency promotion	• Organisations championing cultural competency—encompasses cultural awareness, diversity, and training	[47]
Environment	Multicultural agenda/policies	Multicultural agenda/policies	• Government policy on workplace diversity	[47]

All of the included studies examined knowledge of SEA immigrant-specific health- and sociocultural-related factors 44–50. Six of seven studies discussed HCPs’ belief that inadequate immigrant-specific support influences optimal healthcare provision^44^, ^45^, ^47^–^50^ . Less than half of the studies described immigrant-specific practice 47, 48, 50.

According to HCPs, healthcare practice barriers were cultural discordance with patients and acculturation with the host country; a gap in HCPs’ cultural understanding, communication, and clinical skills; the organisation’s limited resources tailored for HCPs and patients; and funding constraints from the host country’s health funding body. Facilitators identified by HCPs were the patient’s community and health provider support, the HCP’s awareness and desire to provide immigrant-specific care, the organisation’s culturally appropriate services and resources, and the country’s multicultural agendas and policies.

#### HCP Knowledge, Attitudes, and Practices

HCPs demonstrated knowledge of SEA immigrants’ cultural and health beliefs, family and community influence, communication challenges, and specific healthcare needs [45–50]. HCPs were aware of the availability of interpreter services and logistical challenges in providing support to a multicultural community [47, 49, 50], including inadequate training of interpreters in healthcare delivery and of HCPs on immigrant patients’ unique health needs associated with cultural and health beliefs, and migration experiences [46–48, 50]. HCPs also acknowledged the inadequate cultural and ethnic diversity in the workplace [47, 50].

HCPs believed that the inadequate support and funding specifically tailored to providing care to immigrant communities influenced their ability to deliver appropriate care [44, 47–50]. HCPs were concerned and frustrated by their lack of understanding of patients’ cultural beliefs, knowing that these beliefs influence patients’ self-care and health service engagement [45, 47, 48, 50]. However, despite the discordance and distrust, HCPs felt that some patients appreciated and accepted concepts of Western medicine [45, 47, 48, 50].

In practice, HCPs worked with formal interpreters or family members to address the language barrier and act as liaisons with health services [47, 50]. Notably, the cultural beliefs and behaviours of immigrant HCPs influenced their communication style and provision of care to patients of similar backgrounds [48].

#### Patient-Level Barriers and Facilitators

According to HCPs, the clinical interactions with SEA immigrants were influenced by health beliefs, cross-cultural communication barrier, limited literacy, and the patient’s socioeconomic and sociocultural context. In nearly all studies, HCPs identified discordance in health beliefs between the provider and the patient as a barrier to delivering healthcare [44–48, 50]. HCPs found it challenging when patients have a different understanding of cardiometabolic syndrome compared to the Western models of medicine [46–48, 50]. Renfrew et al. (2013) reported that HCPs were finding it difficult to understand if patients perceived diabetes as a disease or a curse and how diabetes is described in their language. HCPs described some SEA-born patients perceived diabetes to come from excess heat in the body; thus, patients were wary of Western pharmaceuticals, which were considered ‘hot’ and laden with undesirable chemicals [48]. This often led to medication hesitancy and avoidance, especially insulin use [45, 47, 48, 50]. The mismatch between Western medicine concepts extended to patients’ dietary habits. For example, HCPs observed the lack of awareness of carbohydrates as a type of macronutrient in some cultures leading to a focus on avoiding only sweet foods and/or calorie restriction [44, 50]. Additionally, HCPs reported that the trauma of mass starvation experienced before migration in some SEA immigrants also potentially led to an inability to limit food intake with ‘binge eating’ observed as a coping mechanism [50]. HCPs might find it challenging to adapt Western medicine concepts to align with the cultural context of the patient. Therefore, confusion and disengagement in managing cardiometabolic syndrome could potentially occur because of health belief differences.

Cross-cultural communication barriers further complicated the discordance between HCPs and patients due to limited English language proficiency and cultural behaviours [44, 46–50]. Communication challenges due to language discordance led HCPs to depend on family members and formal interpreters, which could limit optimal health service delivery [44, 47, 49, 50]. Interestingly, further communication challenges that HCPs identified were cultural behaviour influences [47, 48, 50], as illustrated by the perceived ‘desire to please’ and ‘not disappoint’ the HCP during clinical interactions [50]. Some HCPs suggested that miscommunications or misunderstandings with the patient may stem from differences in cultural beliefs rather than from the patient’s attitude [47]. An additional patient barrier identified by HCPs was limited literacy, which can lead to difficulties in SEA-born patients in understanding and accepting Western medicine concepts, navigating the healthcare system, and accessing health services [46–48, 50]. For example, low levels of literacy could limit receptiveness to new health concepts [46, 48] as well as leading to difficulties in navigating health services for pharmacy prescription refills and pick-ups [50].

HCPs identified that SEA-born patients’ sociocultural and socioeconomic context could pose a barrier to effective care [44–50]. As a result of immigration, changes in family dynamics, including familial care-taking or financial-supporting responsibilities, such as family elders or grandparents becoming primary caregivers of the younger generation [50] or established family members taking on a supporting role to new family immigrants [46], may impede patient engagement with their health needs [46, 50]. According to HCPs, patients’ healthcare engagement could also be influenced by religious beliefs, such as surrendering the fate of their health to their faith and believing it to be God’s will [46]. For some patients, this could lead to withdrawal from personal responsibility for disease self-management and healthcare engagement [46].

HCPs have noted the significant role of the family and community in accessing health services and enhancing patient’s receptivity and understanding of Western health concepts [46, 47, 49, 50]. Family members supported the patient’s access and interaction with HCPs and health services as informal interpreters and liaisons, which is crucial for overcoming language and cultural barriers, while also providing financial and logistical support [46, 47, 50]. The clinical encounters during consultation attendance with HCPs and engagement with health services appeared to facilitate patients’ confidence and knowledge of Western medicine practice [47]. Some HCPs felt that a patient’s confidence is encouraged by their community’s acceptance of Western health concepts [49].

#### Healthcare Team–Level Barriers and Facilitators

At the healthcare team level, HCPs reported the gap in their cultural understanding, communication, and clinical skills as barriers. In contrast, their practice was facilitated by awareness of immigrant-specific needs listed in Tables 3 and 4. HCPs were aware of some of the different health practices of SEA patients and acknowledged their limited knowledge and understanding of non-Western health practices and beliefs [45, 47, 48, 50]. There was a lack of appropriate training regarding the community’s cultural and health needs [47, 50]. According to some HCPs, cultural incompatibility could lead to misunderstandings, such as mistaking cultural issue disagreements for patient attitude [47]. Poor comprehension of patients’ immigration experiences and sociocultural factors [47, 48, 50] were reported to be a barrier to effective care. It is worth noting that the circumstances of immigration are diverse, including escape from conflict, which can result in a lack of patient medical history and severe trauma, therefore presenting significant challenges for HCPs [50]. Additionally, it has been reported that clinical training on cardiometabolic syndrome management for some HCPs was inadequate, for example, on standard health monitoring parameters [48].

HCPs acknowledged and supported the critical role of formal and informal (family) interpreter services in addressing the language gap [47, 50]. However, HCPs pointed out that interpreters could create a ‘sense of distance’ and were inconducive to rapport building between HCPs and patients, compounded by limited consultation time [47, 50]. HCPs also questioned the accuracy of the translated information [47].

Notably, HCPs identifying as immigrants, reflected that their cultural behaviours influenced their practice [48]. For example, aspects such as ‘traditional respect for elders’, as well as the belief that worrying might worsen the condition, could lead to HCPs oversimplifying information and as a result hesitance to discuss other aspects of management [48].

Clinical encounters with immigrant patients and communities facilitated the HCPs’ cultural awareness and knowledge of factors that might affect a patient’s health and management [47, 49]. Cultural awareness was observed more in the studies involving HCPs from similar cultural backgrounds to the patients or HCPs who are engaged with the patient’s culture [45–49].

#### Organisation or Workplace-Level Barriers and Facilitators

This review identified organisational barriers focused on the limited resources tailored for HCPs and SEA immigrant patients [44, 47, 50]. For instance, HCPs voiced that allowances for the additional time required for interpretation were not included in the workflow of some organisations [47, 50]. In addition, interpreter services that catered to patients’ specific needs, such as for ‘after-hours consultations, the elderly, gender-sensitive and extended to relatives’, were inadequate [47]. The availability of bilingual HCPs that could address language discordance was limited [47]. HCPs suggested that the health services and resources provided for immigrants by the organisation might not match the needs and expectations of some patients, such as when translated resources were too basic and simple [44] or when the cultural health perspectives were not incorporated [47]. Conversely, HCPs identified partnerships with the patient’s family and/or community might facilitate the delivery of appropriately tailored health services and could provide support in implementing healthcare activities [47].

#### Environment-Level Barriers and Facilitators

At the environmental level, HCPs identified funding constraints for tailored healthcare services, such as extended appointment times and interpreter services, as a significant barrier [47–49]. HCPs reported that the cost of disease management if accessed outside the usual funding scheme could be absorbed by the organisation or patient [47, 48]. HCPs identified that government agenda and policy towards workplace diversity could support better health outcomes for immigrant communities through positive encounters with, and active participation in, the healthcare system [47].

## Discussion

This scoping review aimed to examine and map what is known about HCPs’ knowledge, attitudes, and practices of cardiometabolic syndrome care among SEA immigrants. All studies examined knowledge of SEA immigrant health and sociocultural factors. Most studies discussed HCPs’ views that inadequate immigrant-specific support can impact optimal care. Less than half of the studies reported on immigrant-specific practice. Using the 'Four-Level Model of Healthcare System', healthcare practice barriers according to HCPs were cultural discordance with patients and acculturation with the host country (patient level); gaps in HCPs’ cultural understanding, communication, and clinical skills (healthcare team level); the organisation’s limited resources tailored for HCPs and patients (organisation level); and funding constraints from the host country’s health funding body (environment level). Facilitators identified by HCPs were the patient’s community and health provider support (patient level), the HCP’s awareness and desire to provide immigrant-specific care (healthcare team level), the organisation’s culturally appropriate services and resources (organisation level), and the country’s multicultural agendas and policies (environment level). Consistent with previous literature, HCPs found challenges in providing care for immigrant patients at the different levels of the healthcare system, which were influenced by the patient’s unique sociocultural and socioeconomic background and the host country’s healthcare paradigm [12, 13, 29, 31, 32, 51]. The interdependence of the four levels is essential for understanding the complex dynamics of healthcare service provision for immigrants. In this review, barriers and facilitators at the individual patient, organisation-wide, and environment level were derived from HCPs' insights based on their clinical practice experiences and encounters with the immigrant community. These insights reflect their inseparable nature and the required integrated approach to understanding the multifaceted barriers and facilitators affecting immigrant healthcare.

### HCP Knowledge of Health and Cultural Beliefs

Cardiometabolic syndrome management, including diabetes, requires life-enhancing changes and self-care engagement for optimal health outcomes. Barriers to optimal health services access in this review were consistent with a previous review among ethnic minorities, mainly from South America and Asia, as well as African Americans residing in the USA, Canada, UK, Germany, and Scandinavian countries [31]. The systematic review of 54 studies found cultural discordance and low level of acculturation with the host country, including health and cultural beliefs, a potential barrier at the patient level [31]. Concurrently, the limited understanding and knowledge of sociocultural factors and health beliefs among HCPs may hinder their ability to effectively relate to and address the health needs of the SEA immigrant population [11].

The findings of this scoping review suggest that HCPs’ practice became challenging when the health and cultural beliefs of SEA immigrants did not align with Western medicine concepts [45, 47, 48, 50]. This was particularly challenging when medical dietary recommendations did not conform with consumption habits [31, 44, 45, 47], thus affecting a patient’s relationships with family and community [44, 46, 50]. The cultural relationship to food and the influence of food on community dynamics are consistent with the Asian ‘small feast’ culture [52] and among South Asians [29], where it is considered impolite and potentially offensive not to finish food served. Some immigrants may have stoic cultural beliefs or attitudes of enduring hunger and deprivation to manage diabetes [44], which could pose a risk of severe health consequences. In contrast, a review of lifestyle disease health beliefs among South Asian immigrants in the UK reported a discordance in the cultural view of excess body weight as an indication of good health rather than as a cardiometabolic syndrome risk [29].

Medication use and adherence can be challenging, mainly when patients are unfamiliar with and have contrasting concepts of their condition and Western medicine management [31, 45, 47, 48, 50]. In a recent systematic review of refugees’ health beliefs in developed countries, a lack of knowledge and understanding of diabetes among Middle Eastern and Yugoslavian immigrants when compared with locally-born participants was found [53]. Immigrant patients were sceptical of the benefits of health services [31]. The lack of confidence and distrust of immigrant patients towards Western medicine identified in this review [50] aligns with findings from a systematic review on the impact of personal and cultural beliefs on medication adherence [30].

Knowledge of cultural practices, beliefs, and values allows HCPs to tailor health interventions to better fit the patients’ lifestyles and cultural contexts [21]. When patients feel understood and respected, they are more likely to engage actively in their care, leading to better management of chronic conditions and overall health outcomes [14, 22].

### HCP Attitudes, Behaviours, and Communication Approach

The knowledge and understanding of a medical condition are paramount to patient engagement with self-management and health services, and to optimal health outcomes [15]. Language barriers in providing care among SEA immigrants can pose significant challenges to achieving this goal [44, 47, 49, 50]. But beyond the language barrier, HCP’s attitudes, behaviours, and communication approach are important determinants of patient self-care engagement [14].

HCPs’ attitudes and behaviours in communication with migrant and minority patients significantly impact the quality of care and health outcomes. Positive attitudes and culturally sensitive behaviours can foster trust and rapport [21, 23], supporting patients to more likely engage with treatment goal and self-care [13, 22]. A review of HCPs involved in the care of immigrants from Asia, Africa, Eastern and South-eastern Europe, Latin America, and the Pacific region proposed that the perceptions, attitudes, and practices of HCPs were influenced by language differences and diverse cultural beliefs, compounded by limited organisational time and resources [12]. Such findings indicate consensus that optimal health management is hindered by language discordance between HCPs and their patients [12, 13, 29, 31–33, 51]. The gap in effective communication was frustrating for HCPs, hindering their ability to provide care that could support patients’ genuine understanding and promote engagement in self-care [47, 50]. Patients would seek HCPs who speak the same language for better communication, bypassing the need for translator services [49]. Patients believed that HCPs of similar cultural and linguistic backgrounds were more likely to understand their needs [22].

Of note, language translation services, usually involving family members, can bridge the communication gap, but the involvement of formal interpreters could potentially result in miscommunication and power dynamic imbalances [44, 47, 49, 50]. This finding is consistent with previous literature on HCPs’ views of the critical role of formal interpreters but questions their accuracy and trustworthiness, particularly in interpreting medical information [32, 33]. The challenge in communication might also result from limited literacy, such that patients may not fully understand the concept and reason for the treatment, ultimately placing pressure on family members to assist with managing the patients’ conditions [44, 50]. Family members are more involved by default in the decision-making process when acting as informal interpreters [32, 54]. The systematic review on family members’ involvement during treatment decision-making in chronic diseases reported that this could lead to feelings of burden and isolation, family tension, and risk of depression [54].

This review also highlighted that cultural behaviours could influence the communication skills of both patients and HCPs; for example, patients’ deference and desire to please the provider might limit communication reliability [50]. This follows findings from previous studies where immigrant patients tended to view HCPs in high regard; thus, asking HCPs questions or asserting personal views was uncommon [28, 29, 31] unless prompted by the HCPs [31, 33]. HCPs also reported adopting a more directive and simplified communication style, often withholding information from immigrant patients due to perceived language barriers and prolonged consultation time due to interpreting [12, 33]. HCPs’ direct and simplified communication due to the language barrier is also reported from a systematic review of South Asian immigrants’ experience with patient-centred care [22].

Effective communication by HCPs is essential to understanding and managing immigrant patients’ health conditions. Improving HCPs’ attitudes and behaviours toward culturally sensitive communication can bridge health disparities and enhance health outcomes for patients.

### Health Service Practices

At the organisational and environmental levels, this review identified the importance of considering the patient’s needs and context in funding and producing healthcare services [44, 47, 48, 50]. This perspective aligns with one of the factors in collaboratively creating healthcare services with immigrants, building on the patient’s narrative and/or solution for the patient’s situation [55]. These narratives may include language barriers, geographical region of origin, family dynamics, marital status, education level, occupation, and social class [31].

Most of the studies identified the lack of appropriate interpreters and additional time, likely due to funding constraints, contributed to health disparities in providing healthcare [44, 47, 49, 50], which was consistent with previous reviews on healthcare services among immigrants [12, 13, 28, 33]. Interestingly, this review found that simplifying translated health resources [48, 50] might not be appropriate for some immigrant populations, suggesting that low English proficiency only sometimes relates to poor health literacy [44]. Translated resources must consider the different values regarding health, idiosyncratic expressions, and levels of literacy among immigrants [31].

Another significant context of the immigrant patient narrative was family dynamics, including familial care, taking responsibility, and financial support obligations [46, 50]. This finding reflects the collectivist nature of the SEA immigrants’ relationship with their community. In a 2020 review of diabetes and hypertension intervention among Filipino-Americans, engagement and partnership with patients’ community (professional, non-profit, or faith-based organisation) and inclusion of family and community in the activities were found to improve service recruitment, retention, and completion [26]. Among South Asian immigrants in the UK, the importance of community endorsement and inclusion of the family or household in implementing behavioural lifestyle changes were another example of the collectivist nature of some immigrant groups [29]. The literature highlights the significance of involving the patient’s community to improve their management of cardiometabolic syndrome, as patients often reach out to their community for support, education, and advice and might not see the need to interact with formal health services [29, 31]. Rather than only emphasising the promotion of individual health, health services should include initiatives to promote family and social support and community empowerment to adopt healthy lifestyles [27, 29]. Other initiatives to promote health outcomes reported in previous studies that can be adopted by HCPs when providing care to SEA immigrants with cardiometabolic syndrome include implementing culturally sensitive interventions, for example, having bilingual community health workers who were members of the local community to deliver culturally sensitive and appropriate health education programmes [19, 27].

Immigrant HCPs included in this review were found to adjust their practice to accommodate cultural health beliefs, specifically upholding ‘traditional respect of elders’, which could involve not pursuing treatment optimisation that the patient finds stressful or upsetting [48]. Such observation has been reported previously by Scheppers et al. (2006), where the clinical practice behaviours of HCPs with similar ethnic or cultural backgrounds to patients were influenced by social class, education, gender identification, or generation differences between patients and HCPs [31]. However, the authors further argued that any cultural congruence between HCPs and patients could be negated by differences in clinical reality between the HCP’s objective goal of diagnosis and treatment and the patient’s subjective experience of living the condition [31].

Consistent with previous literature on the perceptions, attitudes, and practices of HCPs involved in the care of immigrants from Asia, Africa, Eastern and South-eastern Europe, Latin America, and the Pacific region, cultural and communication challenges interfered with providing holistic care [12] and the HCPs’ role as advisors and collaborators in the patient’s self-care engagement. Similar to Robertshaw et al.’s 2017 systematic review on HCP challenges in providing primary healthcare for immigrants, including Afghan, African, Cambodian, East Timorese, Kosovars, Somali and Vietnamese immigrants residing in the USA, Canada, UK, Switzerland, Australia, New Zealand, and Scandinavian countries, this scoping review also identified the key challenges of limited HCPs communication skill and resources, limited cultural understanding, unique healthcare needs of immigrants, limited diversity and cultural encounters of HCPs and organisation, and inadequate funding [32]. The barriers in this review identified by HCPs from the different healthcare system levels on cultural discordance, including cross-cultural communication challenges, unmet needs of the immigrant community, and limited diversity in health services, may add additional evidence to the potential universality of barriers to patient health services engagement.

### Integrated Health System

Immigrants from SEA brought their cultural and health beliefs and behaviours, which might differ from what HCPs have previously encountered. If HCPs have a broader understanding of the cultural backgrounds of the population, they may be able to adapt their counselling to the dynamic health beliefs and priorities of the patient. This understanding and awareness were facilitated by ongoing encounters that allowed for the process of learning between the providers and the patients [47, 49, 50] and the shared cultural backgrounds of providers and patients [45–49]. Concordance or mutual understanding between HCPs and patients from the outset and a shared approach to care facilitated access to culturally appropriate health services [34].

In this review, facilitators of cultural encounters from the different healthcare system levels could be effectively implemented by involving all stakeholders, especially the patients/consumers. Using the 'Four-Level Model of Healthcare System', the environment (governing body) could set the stage and tone of the healthcare services with policies, standards, and funding; the organisation could provide culturally appropriate services and resources and workforce diversity to support the needs of the HCPs and patients; the clinical practice of the healthcare team could be in a constant state of learning and adaptation to the nuanced needs of the patient; and the patient and their community as active partners of the health system in co-producing the health services to meet their healthcare needs (see Fig. 1).

Co-production is an approach to co-creating health services from the collaborative relationship between patients/consumers and HCPs [55, 56]. Co-production with immigrant patients could address barriers to service access and delivery and improve efficiency and health equity [55]. It has been proposed that co-production between patients and HCPs is interconnected with the different levels of the healthcare system [56]. This interconnectivity and influence are not constrained within the healthcare system. They could extend to social forces and other services in the broader community [56], further reinforcing the importance of community involvement in healthcare and supporting HCPs KAP in optimally providing care to patients from diverse cultural backgrounds.

This review has several limitations that should be considered when interpreting the results. This scoping review included a small number of studies (*n* = 7) involving the perspectives of HCPs in developed countries. Their interactions were limited to immigrants from five of the ten member countries of ASEAN. Hence, its applicability may potentially only be extrapolated to developed countries and to immigrants from the included countries. Nearly all included papers were published more than a decade ago which may not capture the changes in cross-cultural healthcare practice [57]. While the population group in the search strategy included patients and consumers, their perspectives were not included. All results were presented from the HCPs’ point of view. In addition, some of the included papers had limited exploration of HCPs’ perspectives and experiences. One notable limitation of this scoping review is that previous researchers have already interpreted the data analysed. Consequently, the findings included in our review inherently reflect the perspectives and interpretations of those researchers, potentially introducing bias or subjective viewpoints into the synthesised data. This secondary analysis limits our ability to independently verify or re-interpret the raw data, which may affect the objectivity and reliability of our conclusions. Recognising this limitation is crucial for contextualising our findings and understanding the potential influence of prior researchers’ perspectives on the results presented in this review. It is also noted that most of the papers included in this review identified HCP participants who were of similar cultural [45, 46, 48, 49] and immigrant [47] backgrounds that might have enabled the identification of the culturally related barriers and facilitators. This review excluded undocumented migrants [3]; thus, the barriers encountered by this group were not reported. Limiting English-language studies may also exclude potential studies in other languages that meet the inclusion criteria.

## Conclusion

This scoping review has highlighted the barriers and facilitators encountered by HCPs’ KAP in providing healthcare for SEA immigrants with cardiometabolic syndromes, as well as the similarities also encountered by other HCPs caring for other immigrant groups. The findings advocate for future research on adopting co-production in healthcare service design to support HCPs’ KAP. The active involvement of patients, their families, and communities is paramount to ensure services are culturally congruent and enhance the accessibility and appropriateness of health services for diverse populations. The integrated approaches can support HCPs in providing culturally appropriate care and achieving health equity for all members of society, regardless of their ethnic or cultural background.

## Supplementary Information

Below is the link to the electronic supplementary material.

Supplementary file1 (DOCX 27 KB)

Supplementary file2 (DOCX 18.4 KB)

## Data Availability

The data supporting this scoping review's findings are derived from publicly available sources, which are cited in the paper. The extracted data, including the list of articles reviewed and any other supplementary materials, are available from the corresponding author upon request. As this is a review paper, no new primary data were generated during this study.
