# Supplementary material for: Healthcare Professionals’ Knowledge, Attitudes, and Practices in Providing Care to Southeast Asian Immigrants with Cardiometabolic Syndrome: A Scoping Review
Source: J Racial Ethn Health Disparities. 2024 Aug 20;12(5):3253–68. doi: 10.1007/s40615-024-02129-3 (PMC12446144; doi:10.1007/s40615-024-02129-3)
Supplement: Supplementary file 2 — Supplementary file2 (DOCX 18.4 KB) [file 40615_2024_2129_MOESM2_ESM.docx]

**Appendix 2**

Scoping review search strategy

| **DATABASE** | **Search Date** | **Hits** | **LIMITS or Exclusion** | **Fields for All Search Terms** |
| --- | --- | --- | --- | --- |
| Embase | 17/07/2023 | 394 | None | explode |
| PubMed | 17/07/2023 | 125 |  | All Fields, Mesh Terms |
| CINAHL | 17/07/2023 | 67 |  | Expanders - Apply related words; Also search within the full text of the articles; Apply equivalent subjects |
| PsycINFO | 17/07/2023 | 33 |  | anywhere |

Search strategy using PubMed

| Search  number | Query | Search Details | Results |
| --- | --- | --- | --- |
| 6 | #1 AND #2 AND #3 AND #4 AND #5 | | 125 |
| 5 | "southeast asia*" OR "south east asia*" OR cambodia* OR indonesia* OR laos OR laotian OR malaysia* OR singapore* OR filipino OR Philippines OR thai* OR vietnam* OR burm* OR myanma* OR brunei* | "southeast asia*"[All Fields] OR "south east asia*"[All Fields] OR "cambodia*"[All Fields] OR "indonesia*"[All Fields] OR "laos"[MeSH Terms] OR "laos"[All Fields] OR "laotian"[All Fields] OR "laotians"[All Fields] OR "malaysia*"[All Fields] OR "singapore*"[All Fields] OR "filipino"[All Fields] OR "filipinos"[All Fields] OR "philippine"[All Fields] OR "philippines"[MeSH Terms] OR "philippines"[All Fields] OR "thai*"[All Fields] OR "vietnam*"[All Fields] OR "burm*"[All Fields] OR "myanma*"[All Fields] OR "brunei*"[All Fields] | 487,300 |
| 4 | immigrant OR migrant OR emigrant OR refugee OR "ethnic minorit*" OR "vulnerable population" OR "minority group" OR "health minorit*" OR "culturally and linguistically diverse" OR CALD | "emigrants and immigrants"[MeSH Terms] OR ("emigrants"[All Fields] AND "immigrants"[All Fields]) OR "emigrants and immigrants"[All Fields] OR "immigrant"[All Fields] OR "immigrants"[All Fields] OR "emigration and immigration"[MeSH Terms] OR ("emigration"[All Fields] AND "immigration"[All Fields]) OR "emigration and immigration"[All Fields] OR "immigration"[All Fields] OR "immigrations"[All Fields] OR "immigrant s"[All Fields] OR "immigrate"[All Fields] OR "immigrated"[All Fields] OR "immigrates"[All Fields] OR "immigrating"[All Fields] OR ("migrant s"[All Fields] OR "transients and migrants"[MeSH Terms] OR ("transients"[All Fields] AND "migrants"[All Fields]) OR "transients and migrants"[All Fields] OR "migrant"[All Fields] OR "migrants"[All Fields]) OR ("emigrants and immigrants"[MeSH Terms] OR ("emigrants"[All Fields] AND "immigrants"[All Fields]) OR "emigrants and immigrants"[All Fields] OR "emigrant"[All Fields] OR "emigrants"[All Fields] OR "emigrate"[All Fields] OR "emigrated"[All Fields] OR "emigrates"[All Fields] OR "emigrating"[All Fields] OR "emigration and immigration"[MeSH Terms] OR ("emigration"[All Fields] AND "immigration"[All Fields]) OR "emigration and immigration"[All Fields] OR "emigration"[All Fields] OR "emigrations"[All Fields] OR "emigres"[All Fields]) OR ("refugee s"[All Fields] OR "refugees"[MeSH Terms] OR "refugees"[All Fields] OR "refugee"[All Fields]) OR "ethnic minorit*"[All Fields] OR "vulnerable population"[All Fields] OR "minority group"[All Fields] OR "health minorit*"[All Fields] OR "culturally and linguistically diverse"[All Fields] OR "CALD"[All Fields] | 125,103 |
| 3 | hypertension OR "high blood pressure" OR "elevated blood pressure" OR hypertensive OR "high cholesterol" OR "high sugar" OR "cardiometabolic risk factors" OR "metabolic syndrome" OR diabetes OR prediabet* OR "impaired glucose tolerance" OR "impaired fasting glucose" OR "insulin resistance" OR "abdominal obesity" OR "cardiovascular disease" OR "cardiac disease" OR "cardiovascular health" OR "heart disease" OR "heart condition" OR "heart disease risk factors" OR hyperlipidemia OR hyperlipidaemia OR dyslipidemia OR dyslipidaemia OR hypercholesterol*emia OR "lipid disorder" | "hypertense"[All Fields] OR "hypertension"[MeSH Terms] OR "hypertension"[All Fields] OR "hypertension s"[All Fields] OR "hypertensions"[All Fields] OR "hypertensive"[All Fields] OR "hypertensive s"[All Fields] OR "hypertensives"[All Fields] OR "high blood pressure"[All Fields] OR "elevated blood pressure"[All Fields] OR ("hypertense"[All Fields] OR "hypertension"[MeSH Terms] OR "hypertension"[All Fields] OR "hypertension s"[All Fields] OR "hypertensions"[All Fields] OR "hypertensive"[All Fields] OR "hypertensive s"[All Fields] OR "hypertensives"[All Fields]) OR "high cholesterol"[All Fields] OR "high sugar"[All Fields] OR "cardiometabolic risk factors"[All Fields] OR "metabolic syndrome"[All Fields] OR ("diabete"[All Fields] OR "diabetes mellitus"[MeSH Terms] OR ("diabetes"[All Fields] AND "mellitus"[All Fields]) OR "diabetes mellitus"[All Fields] OR "diabetes"[All Fields] OR "diabetes insipidus"[MeSH Terms] OR ("diabetes"[All Fields] AND "insipidus"[All Fields]) OR "diabetes insipidus"[All Fields] OR "diabetic"[All Fields] OR "diabetics"[All Fields] OR "diabets"[All Fields]) OR "prediabet*"[All Fields] OR "impaired glucose tolerance"[All Fields] OR "impaired fasting glucose"[All Fields] OR "insulin resistance"[All Fields] OR "abdominal obesity"[All Fields] OR "cardiovascular disease"[All Fields] OR "cardiac disease"[All Fields] OR "cardiovascular health"[All Fields] OR "heart disease"[All Fields] OR "heart condition"[All Fields] OR "heart disease risk factors"[All Fields] OR ("hyperlipidaemia"[All Fields] OR "hyperlipidemias"[MeSH Terms] OR "hyperlipidemias"[All Fields] OR "hyperlipidemia"[All Fields] OR "hyperlipidaemias"[All Fields]) OR ("hyperlipidaemia"[All Fields] OR "hyperlipidemias"[MeSH Terms] OR "hyperlipidemias"[All Fields] OR "hyperlipidemia"[All Fields] OR "hyperlipidaemias"[All Fields]) OR ("dyslipidaemias"[All Fields] OR "dyslipidemias"[MeSH Terms] OR "dyslipidemias"[All Fields] OR "dyslipidaemia"[All Fields] OR "dyslipidemia"[All Fields]) OR ("dyslipidaemias"[All Fields] OR "dyslipidemias"[MeSH Terms] OR "dyslipidemias"[All Fields] OR "dyslipidaemia"[All Fields] OR "dyslipidemia"[All Fields]) OR "hypercholesterol*emia"[All Fields] OR "lipid disorder"[All Fields] | 1,857,241 |
| 2 | knowledge OR attitude OR practice OR "health service" OR "health care delivery" OR "delivery of health care" OR "quality care" OR "patient-centred care" OR "patient-centered care" OR "shared decision-making" OR "dietetic care" OR communication OR experience OR view* OR opinion OR perception OR belief OR perspective OR strateg* OR approach | "knowledge"[MeSH Terms] OR "knowledge"[All Fields] OR "knowledge s"[All Fields] OR "knowledgeability"[All Fields] OR "knowledgeable"[All Fields] OR "knowledgeably"[All Fields] OR "knowledges"[All Fields] OR "attitude"[MeSH Terms] OR "attitude"[All Fields] OR "attitudes"[All Fields] OR "attitude s"[All Fields] OR "practicability"[All Fields] OR "practicable"[All Fields] OR "practical"[All Fields] OR "practicalities"[All Fields] OR "practicality"[All Fields] OR "practically"[All Fields] OR "practicals"[All Fields] OR "practice"[All Fields] OR "practice s"[All Fields] OR "practiced"[All Fields] OR "practices"[All Fields] OR "practicing"[All Fields] OR "health service"[All Fields] OR "health care delivery"[All Fields] OR "delivery of health care"[All Fields] OR "quality care"[All Fields] OR "patient-centred care"[All Fields] OR "patient-centered care"[All Fields] OR "shared decision-making"[All Fields] OR "dietetic care"[All Fields] OR "communicate"[All Fields] OR "communicated"[All Fields] OR "communicates"[All Fields] OR "communicating"[All Fields] OR "communication"[MeSH Terms] OR "communication"[All Fields] OR "communications"[All Fields] OR "communicative"[All Fields] OR "communicational"[All Fields] OR "communicatively"[All Fields] OR "communicativeness"[All Fields] OR "communicator"[All Fields] OR "communicator s"[All Fields] OR "communicators"[All Fields] OR "experience"[All Fields] OR "experience s"[All Fields] OR "experiences"[All Fields] OR "view*"[All Fields] OR "attitude"[MeSH Terms] OR "attitude"[All Fields] OR "opinion"[All Fields] OR "opinions"[All Fields] OR "opinion s"[All Fields] OR "opinionated"[All Fields] OR "percept"[All Fields] OR "perceptibility"[All Fields] OR "perceptible"[All Fields] OR "perception"[MeSH Terms] OR "perception"[All Fields] OR "perceptions"[All Fields] OR "perceptional"[All Fields] OR "perceptive"[All Fields] OR "perceptiveness"[All Fields] OR "percepts"[All Fields] OR "belief s"[All Fields] OR "culture"[MeSH Terms] OR "culture"[All Fields] OR "belief"[All Fields] OR "beliefs"[All Fields] OR "perspective"[All Fields] OR "perspective s"[All Fields] OR "perspectives"[All Fields] OR "strateg*"[All Fields] OR "approach"[All Fields] OR "approach s"[All Fields] OR "approachability"[All Fields] OR "approachable"[All Fields] OR "approache"[All Fields] OR "approached"[All Fields] OR "approaches"[All Fields] OR "approaching"[All Fields] OR "approachs"[All Fields] | 9,004,895 |
| 1 | "health* personnel" OR "health personnel" OR "primary healthcare" OR "health professional" OR "healthcare provider" OR "health practitioner" OR diet* OR nutritionist OR pharmac* OR dent* OR physician* OR clinician OR "general practitioner" OR doctor OR nurse OR optometr* OR podiatrist OR therapist OR "diabetes educator" OR psycho* OR "family practice" OR "multidisciplinary care team" OR "health care" OR "primary care" OR "shared services" OR "medical interpreter" OR patient* OR consumer* | "health personnel"[All Fields] OR "health personnel"[All Fields] OR "primary healthcare"[All Fields] OR "health professional"[All Fields] OR "healthcare provider"[All Fields] OR "health practitioner"[All Fields] OR "diet*"[All Fields] OR "nutritionist s"[All Fields] OR "nutritionists"[MeSH Terms] OR "nutritionists"[All Fields] OR "nutritionist"[All Fields] OR "pharmac*"[All Fields] OR "dent*"[All Fields] OR "physician*"[All Fields] OR "clinician"[All Fields] OR "clinician s"[All Fields] OR "clinicians"[All Fields] OR "general practitioner"[All Fields] OR "doctor s"[All Fields] OR "doctoral"[All Fields] OR "doctorally"[All Fields] OR "doctorate"[All Fields] OR "doctorates"[All Fields] OR "doctoring"[All Fields] OR "physicians"[MeSH Terms] OR "physicians"[All Fields] OR "doctor"[All Fields] OR "doctors"[All Fields] OR "nurse s"[All Fields] OR "nurses"[MeSH Terms] OR "nurses"[All Fields] OR "nurse"[All Fields] OR "nurses s"[All Fields] OR "optometr*"[All Fields] OR "podiatrist"[All Fields] OR "podiatrist s"[All Fields] OR "podiatrists"[All Fields] OR "therapist"[All Fields] OR "therapist s"[All Fields] OR "therapists"[All Fields] OR "therapists s"[All Fields] OR "diabetes educator"[All Fields] OR "psycho*"[All Fields] OR "family practice"[All Fields] OR "multidisciplinary care team"[All Fields] OR "health care"[All Fields] OR "primary care"[All Fields] OR "shared services"[All Fields] OR "medical interpreter"[All Fields] OR "patient*"[All Fields] OR "consumer*"[All Fields] | 16,857,046 |
